# Editorial: How do behavior science interventions to reduce environmental impacts work in the real world?

**DOI:** 10.3389/fpsyg.2025.1696849

**Published:** 2025-11-03

**Authors:** Christian Andreas Klöckner, Giuseppe Carrus, Jens Røyrvik, Michael Niederkofler

**Affiliations:** ^1^Department of Psychology, Norwegian University of Science and Technology, Trondheim, Norway; ^2^Department of Educational Science, University Roma Tre, Rome, Italy; ^3^Department of Anthropology, Norwegian University of Science and Technology, Trondheim, Norway; ^4^Innovation Lab act4energy, Stegersbach, Austria

**Keywords:** behavior science, intervention—behavioral, real world, psychology, environmental behavior

## 1 Introduction

Environmental psychology and related behavior sciences have made substantial progress in theory development and in studying interventions to change people's pro-environmental behavior, as demonstrated by numerous meta-analyses published over the past decades (e.g., Bamberg and Möser, 2007; Klöckner, 2013; Maki et al., 2019; Udall et al., 2021; Osbaldiston and Schott, 2012). However, environmental psychologists have also been encouraged to place greater emphasis on the potential impact of the behaviors they aim to change and to invest more effort into developing and testing interventions targeting such high-impact behaviors (e.g., Nielsen et al., 2021; Gatersleben, 2023), while at the same time not ignoring the role of smaller or symbolic actions in signaling changing social norms and building momentum for broader change. Furthermore, although environmental psychology thrives as a discipline, the strong dominance of studies conducted in Western cultures is increasingly criticized within psychology in general, including in environmental psychology (e.g., Gould et al., 2025; Masuda et al., 2020). Montesanti et al. (2021) provide a good example of how incorporating indigenous perspectives on coping with environmental stress can enrich theory building and practices of dealing with real-world issues (wildfires, in this case). Finally, several researchers have repeatedly called for a stronger focus of environmental psychology on the usability of its research for practical implementation (e.g., Ernst and Wenzel, 2014; Clayton et al., 2016). We wholeheartedly support these calls for a behavior science that makes a real difference by focusing on behaviors with the potential to change impact substantially, extending beyond the WEIRD populations and providing clear recommendations to practitioners. Combining the last two points, recommendations to practitioners beyond the Western world remain largely neglected in environmental psychology. In our view, this also requires a focus on articles studying the effects of psychological interventions in the real world. Taking this as a starting point, we launched our call for a Research Topic in 2024 with exactly this focus, the outcomes of which are presented here.

## 2 Focusing on impact in the real world

To be able to make a change in the real world, behavior science needs to study behaviors that have the potential to make a difference, when changed, either because they have a high environmental impact, are practiced by many people, or can stimulate broader changes by establishing new social norms, even if their individual impact is small. Furthermore, behavior change interventions for such behaviors need to be tested under real-world conditions. Within this Research Topic, you will find empirical research that does both: studying relevant behaviors within the complexities of real-world contexts. For example, Wilson and Whitmarsh focus on one of the most impactful categories of individual behavior—mobility—specifically examining shared e-bike use in rural areas. In addition, Bai et al. focus on mobility through their study on the role of emotions in shaping consumer satisfaction among users of alternative fuel vehicles. Ardesch et al. address another high-impact behavior in their intervention study on reducing meat consumption in cafeterias. Klöckner et al. examine how one-stop webshops providing energy counseling and assistance to homeowners increase their ambition for energy renovation projects, potentially unlocking substantial energy savings. Hinn et al. look at energy use in office buildings and explore the psychological context of energy saving. Höpfl et al. investigate different intervention framing approaches to change people's use of washing machines to reduce energy and water consumption. Furthermore, Mitev et al. focus on reducing water consumption in their intervention study with university freshmen, exploring the potential of windows of opportunities when people change their life situation. A related topic is addressed by Vila-Tojo et al., who examine how resistance to decentralized wastewater treatment can be reduced through information and participatory approaches. Tobias et al. use a theoretically well-grounded approach to design and evaluate an anti-littering campaign in public parks in Switzerland. Finally, Schimmelpfeng et al. address a topic related to the effects of environmental change, namely dengue fever and prophylactic treatment of children. Using the psychological framework of the dragons of inaction (Gifford, 2011), they examine the drivers of dengue transmission in Brazil.

## 3 Going beyond the Western world

The articles in our Research Topic cover a diverse range of countries, such as Norway, the USA, Germany, the UK, the Netherlands, Thailand, China, Spain, Sweden, Brazil, and Switzerland, as well as an international database of car information and discussion platforms. Although the majority of the presented articles are still based on data from Western, mostly European, populations, we would like to highlight the articles by Hinn et al., Ma and Chen, and Schimmelpfeng et al. as important contributions that extend the global understanding of behavior research for the environment. Especially, the study by Ma and Chen seems relevant for this aspect, as it explores the role of social influence on pro-environmental behavior within the Chinese context. Schimmelpfeng et al. apply a well-established psychological framework to test its applicability in a Brazilian context, focusing on school children and health behavior. However, we would also like to acknowledge that more studies outside the Global North need to be conducted in environmental psychology, especially with challenging established theoretical frameworks and their applicability in new contexts.

## 4 Providing implementation guidelines

A total of three of our articles are literature reviews, addressing questions relevant for both researchers and practitioners: In her article, Schorn presents an interesting summary of findings from 54 studies on social influence on pro-environmental behavior, especially with a focus on how minorities might change the behavior of a majority group. This article provides helpful guidelines for how to design interventions for environmental activists. Baker et al. also address pro-environmental behavior from a social perspective in their review of the relationship between joint pro-environmental behavior and mental wellbeing, identifying particular potential in behaviors involving interaction with nature. The final review in our Research Topic by Mosca et al addresses the interesting question of whether digital tools can enhance pro-environmental behavior. The authors come to the conclusion that this is still largely an under-researched issue, but they are able to give some recommendations. Closely related to this review article, Fjællingsdal proposes guidelines for how to design and scale up environmental game usage in practical applications. Varni et al. examine the design and success of a campaign based on social media, an approach that was also pursued by Mundt et al. and Biresselioglu and Demir present a very hands-on guideline for how to design and monitor the implementation of behavior science-informed interventions to promote pro-environmental behavior. Haga presents findings on how labeling a lamp as ecological changes the context in a positive way, specifically how people in pictures illuminated by this lamp are perceived more favorably. What is striking, however, is that these implementation guidelines strongly focus on interventions in the Global North; therefore, we need more studies and recommendations focusing specifically on environmental behaviors in the Global South.

## 5 Conclusion

This Research Topic of interesting articles shows how vivid environmental psychology and other behavior science research can be, addressing pressing problems of our societies. Looking at the articles, it is clear that such research can help design interventions that really make a difference, but the reviews also show that further research is needed, especially research that extends beyond Europe and North America and, even more specifically, research on interventions and theory building from a non-Western perspective. We also believe that a closer collaboration between behavioral researchers and practitioners might improve the usability of this research further.
